# Improving Sexual and Gender Minority Cancer Care: Patient and Caregiver Perspectives From a Multi-Methods Pilot Study

**DOI:** 10.3389/fonc.2022.833195

**Published:** 2022-05-06

**Authors:** Miria Kano, Shoshana Adler Jaffe, Stephanie Rieder, Mikaela Kosich, Dolores D. Guest, Ellen Burgess, Ariel Hurwitz, Vernon Shane Pankratz, Teresa L. Rutledge, Zoneddy Dayao, Larissa Myaskovsky

**Affiliations:** ^1^ Department of Internal Medicine, University of New Mexico School of Medicine, Albuquerque, NM, United States; ^2^ University of New Mexico Comprehensive Cancer Center, Albuquerque, NM, United States; ^3^ Department of Obstetrics and Gynecology, University of New Mexico School of Medicine, Albuquerque, NM, United States; ^4^ University of New Mexico School of Medicine, Albuquerque, NM, United States; ^5^ Center for Healthcare Equity in Kidney Disease (CHEK-D), University of New Mexico Health Sciences Center, Albuquerque, NM, United States

**Keywords:** sexual and gender minority cancer, cancer care delivery, cancer health disparities, multi-methods research, lesbian, gay, bisexual and transgender

## Abstract

**Purpose:**

Up to 1 million lesbian, gay, bisexual, and transgender (i.e., sexual and gender minority, SGM) individuals in the United States have histories of cancer. This medically underserved population is diverse, with complex sexualities and gender identities, and distinct health concerns. SGM persons experience disproportionate risks for, and rates of, anal, breast, cervical, colorectal, endometrial, lung, and prostate cancers, in addition to cancers affecting transgender persons who have undergone sex-reassignment. SGM individuals are linked by shared experiences of stigmatization as a minority population for which little cancer research has been conducted. SGM cancer patients frequently report reluctance to seek healthcare, have poorer outcomes following diagnosis, engage in elevated risk behaviors (i.e. smoking and alcohol use) even after cancer diagnosis, have difficulty making emotional adjustment to illness, and experience higher rates of psychological distress. They report less satisfaction with cancer care, deficiencies in patient-centeredness and shared decision-making, gaps in care, and social isolation. Minority stress resulting from experiences of anti-SGM sentiment and discrimination affects cancer patients and their informal cancer caregivers. Our paper presents findings from a pilot study to identify gaps and opportunities to improve cancer care for SGM patients and caregivers at the University of New Mexico Comprehensive Cancer Center.

**Methods:**

Between June 2020 and July 2021, we used a multi-methods research design informed by ecological theory to collect qualitative and quantitative data regarding cancer patient and caregiver quality of life (QoL) and experiences of cancer and survivorship care. We used PROMIS measures distributed *via* REDCap to assess QoL (i.e., fatigue, pain interference, pain intensity, anxiety, depression, emotional support, social isolation, and companionship), and conducted in-depth semi-structured interviews. We recruited 10 SGM cancer patients and 8 heterosexual, cisgender (H/C) patient matches, and their self-identified informal cancer caregivers (n=36, dyad total n=18). Interviews ranged from 1 to 2 hours, were audio-recorded and transcribed for analysis. The study was approved by the University of New Mexico Human Research Protections Office Institutional Review Board.

**Results:**

Results of the PROMIS QoL assessments indicated that SGM patients reported greater anxiety [mean (SD) = 54.5 (8.8)] and depression [mean (SD) = 49.3 (4.8)] than H/C patients [mean (SD)=51.6 (7.5) and 45.4 (6.8) respectively], while heterosexual, cisgender (H/C) patients reported higher fatigue [mean (SD) =52.04 (8.18)] and stronger pain intensity than SGM patients [mean (SD)=48.3 (9.1) and 37.8 (9.1) respectively]. SGM patients reported higher levels of social isolation [mean (SD) = 48.3 (7.3) vs. 42.1 (7.4) for H/C patients, whereas H/C patients reported more emotional support (mean (SD) =57.5 (9.3) vs. 53.0 (6.9)] and companionship [mean (SD) = 55.2 (8.6) vs. 51.5 (11.0)]. SGM and H/C differences in caregiver QoL were most notable with regards to higher levels of fatigue [mean (SD) = 47.1 (6.0) for SGM, and 42.4 (11.5) for H/C] and companionship [mean (SD) = 55.3 (6.0) for SGM, and 50.9 (5.5) for H/C]. Qualitative interviews supported our quantitative results. SGM patients and caregivers articulated experiences of anti-SGM stigma and discrimination contributing to minority stress that influenced their initial cancer care encounters. SGM dyads had more trepidation and/or medical mistrust during initial cancer care encounters when compared to H/C patients and caregivers. SGM patients questioned care that was not culturally responsive to SGM preferences, while H/C patients were more apt to identify gaps in communication and perceived lack of clarity regarding cancer care delivery. Although SGM patients experienced high satisfaction with their cancer care once they developed trust with their providers, they discussed desires to have more direct conversations with their oncologists about their sexual orientation and gender identities and sexual health. All patients and providers in the study (SGM and H/C) appreciated their oncology care teams. All patients and caregivers relied on social networks comprised of friends and family, although SGM patients and caregivers had smaller social networks and relied less on biological family, and single SGM individuals experienced challenges accessing cancer care and struggled with social isolation. We discovered too, that all caregivers, regardless of Sexual Orientation and Gender Identity (SOGI), perceived a lack of support and information pertaining to their loved one’s treatment, side effects and best way to provide care.

**Conclusions:**

This study demonstrates that prior stigmatizing experiences contribute to minority stress and medical mistrust for SGM cancer patients and their informal caregivers across the cancer care experience. Findings point to specific gaps in SGM cancer patient care, including lack of conversation about patient SOGI, inadequate staff and oncology provider SGM specific knowledge and cultural competence/cultural humility training, and insufficient patient supports for those who lack social support during cancer care treatment. Further, this study reveals inadequacies in SGM specific support, and overall support services for informal cancer caregivers. Additional research is required to develop targeted interventions to address minority stress and clinic environment concerns to improve cancer care for SGM patients. Importantly, while there were differences between SGM and H/C experiences of cancer treatment, significant similarities also emerged. Caregiver expressed consensus about the current lack of support and guidance for informal caregivers of cancer patients. Future work should focus on providing caregiver-specific resources in the clinic setting and facilitating support groups for caregivers to network with one another, as well as for tailoring SGM specific caregiver support services. Our findings highlight areas for improving cancer care for the SGM community, as well as a broader population of patients and caregivers.

## 1 Introduction

Sexual and gender minority (SGM) individuals (i.e., lesbian, gay, bisexual, and/or transgender) are a diverse population with complex sexualities and gender identities who are medically underserved and at risk for disparate cancer treatment and survivorship care (1–3) According to the latest Gallup Poll, 5.6% of the U.S. population (4) or 18 million adults identify as SGM. Studies suggest that nearly 1,000,000 of these SGM individuals have histories of cancer (5); and that 106,400 will receive new cancer diagnoses and 33,600 will die of cancer in 2021 (4, 6).

When compared to heterosexual, cisgender (H/C) populations [i.e., those partnered with the opposite sex and whose sex assigned at birth matches their gender identity (7)], SGM persons experience disparate rates of anal, breast, cervical, colorectal, endometrial, lung, and prostate cancers (8). Transgender persons receiving hormone therapy may have higher risks for cancer as well (9). SGM persons exist across all populations, often occupying multiple marginalized identities as ethnic/racial minorities, those with low incomes, and/or rural residents (10). They share experiences of stigmatization and/or discrimination as a population for which little cancer research is conducted, and few cancer interventions are successfully developed (7, 8).

Barriers to sustainable SGM cancer health equity are substantial. At patient levels, studies reveal that SGM cancer patients are reluctant to access care, citing previous discrimination (11); have elevated risk behaviors including smoking and alcohol use even after cancer diagnosis (12); and have difficulty making emotional adjustment to illness (13). Some studies indicate too that SGM cancer patients experience higher rates of psychological distress when recovering (14–16) as they are more likely to experience post-traumatic stress and/or depression (17, 18). For older SGM cancer patients, lack of social support is a critical concern (19–21), as older SGM individuals, particularly bisexual and gay men, have a significantly higher likelihood of living alone, putting them at risk of social isolation (22), diagnosis at later stages of disease, lower quality of life, and poorer cancer survival (21). Due to these complex reasons, SGM cancer patients often report less satisfaction with cancer care, gaps in cancer care, unmet psychosocial needs (23), and deficiencies in patient-centeredness and shared decision-making (24).

Our previous research in primary care settings (25, 26) and that of others in cancer treatment milieus indicate that psychosocial challenges unique to SGM populations, such as “minority stress (27, 28), may compound cancer-related-stress (25, 27, 29) and patient feelings of stigmatization in health care settings. Chronic minority stress can cause SGM Individuals to internalize individuals may internalize anti-SGM attitudes and comments, accept discriminatory actions, endure microaggressions (i.e., subtle verbal and behavioral slights and insults), and come to normalize and anticipate negative experiences. Minority stress compounds for those occupying multiple marginalized social positions (i.e. racial/ethnic minorities, rural residents, the socioeconomically disadvantaged) (30, 31), resulting in 1.5 to 3 times higher rates of behavioral health and substance use disorders than heterosexual adults (32). The compounding effects of minority stress on psychological distress resulting from oncology care can exacerbate cancer health disparities for SGM patients (33–35).

Barriers to equitable SGM cancer care exist at informal cancer caregiving levels as well (36–38). Informal cancer caregivers are individuals who assist patients with domestic tasks associated with daily living. They are unpaid, and spend considerable time assisting patients with clinic visits, managing medication, and assisting with clinical decisions (39). Whereas informal caregivers for H/C cancer patients are typically family members, spouses, or partners, SGM patients more often rely on spouse/partners, friends, and community members, and not biological family due to strained relationships resulting from the patient’s sexuality and/or gender identity (21). As indicated previously, gay men are more likely to be single and live alone which has also been found to affect access to care and caregiving relationships during cancer treatment (40). Although caregiver stress and burnout are recognized as a common complication of treating the chronically or terminally ill (41), caregivers of the SGM community face additional concerns. Studies find that caregivers of SGM patients tend to be younger, racially/ethnically diverse, more likely to have lower incomes, and less likely to be married (21). If they are members of the SGM community, they too may have experienced stigma, prejudice and discrimination contributing to minority stress in healthcare settings.

Improvements to SGM cancer care are often hindered by gaps in knowledge, funding, and leadership support at institutional and oncology provider levels. A 2016 national survey of more than 450 oncologists from 45 cancer centers demonstrated that multilevel factors including: 1) environmental- (i.e., sexual orientation and gender identity data collection, cancer center environment), 2) knowledge- (i.e., staff/provider education and skills), and 3) sociocultural-level barriers (i.e., cultural competence) hinder efforts to reduce SGM cancer disparities (6, 42). Thus, to document gaps and identify opportunities to improve care at institutional-, social- and individual- levels, we conducted a multi-methods pilot, informed by ecological theory (43, 44), comparing the experiences of SGM cancer patients and their self-identified cancer caregivers with those of H/C cancer patient/caregiver dyads receiving care at the University of New Mexico Comprehensive Cancer Center.

In this article, we present findings from the PROMIS [Patient-Reported Outcomes Measurement Information System (45)] validated measures used to provide a quality of life (QoL) snapshot of cancer patients and caregivers in areas of fatigue, pain interference, pain intensity, anxiety, depression, emotional support, social isolation, and companionship. We also discuss results from qualitative interviews, comparing experiences of SGM patient/caregiver dyads with those of H/C dyads, highlighting how SGM patient and caregiver experiences of anti-SGM stigma and discrimination contribute to minority stress and medical mistrust at the onset of their cancer care. We conclude by mapping participant suggestions to improve cancer care using an ecological map to demonstrate ways to address SGM cancer disparities at multiple levels of the oncology care experience, and by describing next steps for development of this pilot research.

## 2 Methods and Materials

### 2.1 Study Design and Overview

Between 12/2020 and 07/2021, we used a multi-methods research design, informed by ecological theory, to assess cancer patient and caregiver QoL and document experiences of cancer and survivorship care. Ecological theory recognizes that cancer care occurs through a series of interdependent interactions at multiple levels and in multiple systems, thereby providing a model through which to consider the ways in which interactions at patient, caregiver, community and cancer center levels informed cancer care (46). Patients and caregivers completed a questionnaire *via* an electronic QoL and demographic survey link in REDCap (47) (Research Electronic Data Capture). We assessed experiences of cancer care through semi-structured interviews. All components of the study were approved by the University of New Mexico Human Research Protections Office Institutional Review Board (HRRC #20-385).

### 2.2 Study Sample

Patients were eligible to participate in the study if they were age 18 or older, English-speaking, and either currently undergoing cancer treatment or diagnosed with cancer in the last 5 years. Informal caregivers were eligible to participate if they were age 18 or older, English-speaking, and identified as providing or having provided unpaid care to a cancer patient recruited for this study. We recruited SGM patients first, and then identified their primary informal cancer caregiver. We then recruited heterosexual, cis-gender patients as comparators to the SGM patients based on sex assigned at birth and cancer type. We consented all participants individually prior to the survey and again for the interview. We compensated participants $100 for completing the survey and interview.

### 2.3 Instruments and Methods

#### 2.3.1 Patient and Caregiver Quality of Life (QoL)

We used PROMIS validated instruments to collect QoL measures focused on physical, mental and social health, see **Table 1**. Physical Health was measured for patients using the Ca Bank V1.0 Fatigue – 54 items assessing self-reported symptoms, from mild subjective feelings of tiredness to an overwhelming, debilitating, and sustained sense of exhaustion; Ca Bank v2.2 Pain-Interference - 35 items assessing pain interference or the degree to which pain limits or interferes with an individual’s physical, mental, and social activities. Three items are unique to CaPS in which cancer specific calibrations were used; and Scale V1.0 Pain-Intensity - 3 items measures pain intensity or severity. This measure includes a 0-to-10 numeric rating scale for pain intensity (45). Mental Health was assessed for both patients and caregivers using the Ca Bank v1.0 Anxiety – a 22 item scale capturing anxiety, a prominent aspect of emotional distress. It contains 2 items unique to CaPS in which cancer specific calibrations were used; and Emotional Distress Anger SF 8a - 8 items capturing anger as a fundamental aspect of emotional distress (45). Social Health was determined for patients and caregivers using Bank v2.0 Social Isolation - 16 items measuring global, physical, mental and social health; Bank v2.0 Emotional Support 8a - 10 items assessing perceived feelings of being cared for and valued as a person; having confident relationships; and SF v2.0 Companionship 6a - 6 items assessing the degree to which respondents have access to companionship (45).

**Table 1:** Quantitative PROMIS validated measures employed for the improving SGM cancer care pilot.

Domain	PROMIS Measure	Description	Surveyed
Physical Health	Ca Bank V1.0 Fatigue	54 items assessing self-reported symptoms, from mild subjective feelings of tiredness to an overwhelming, debilitating, and sustained sense of exhaustion	Patients
	Ca Bank v1.1 Pain-Interference	35 items assessing pain interference or the degree to which pain limits or interferes with an individual’s physical, mental, and social activities. Three items are unique to CaPS in which cancer specific calibrations were used	
	Scale v1.0 Pain-Intensity	3 items measures pain intensity or severity. This measure includes a 0-to-10 numeric rating scale for pain intensity	
Mental Health	Ca Bank v1.0 Anxiety	22 items capturing anxiety, a prominent aspect of emotional distress. It contains 2 items unique to CaPS in which cancer specific calibrations were used	Patients and Caregivers
	Ca Bank v1.0 Depression	30 items capturing depression, a prominent aspect of emotional distress. It contains 7 items unique to CaPS in which cancer specific calibrations were used	
	Emotional Distress/Anger SF 8a	8 items capturing anger as a fundamental aspect of emotional distress	
Social Health	Bank v2.0 Social Isolation	16 items measuring global, physical, mental and social health	Patients and Caregivers
	Bank v2.0 Emotional Support 8a	10 items assessing perceived feelings of being cared for and valued as a person; having confident relationships	
	SF v2.0 Companionship 6a	6 items assessing the degree to which respondents have access to companionship	

PROMIS measures and descriptions are available at https://www.healthmeasures.net/explore-measurement-systems/promis/obtain-administer-measures.

Content experts developed PROMIS-Cancer measures (PROMIS-Ca) following review of the adult PROMIS item banks (45) PROMIS measures have been validated across multiple clinical populations, including patients with back pain, cancer, chronic heart failure, chronic obstructive pulmonary disease, major depressive disorder, osteoarthritis, and premenstrual syndrome (48, 49). Although not used extensively in SGM focused cancer studies, PROMIS measures have been used successfully to assess and compare disparities related to QoL between heterosexual, lesbian, and bisexual women cancer survivors (50).

#### 2.3.2 Characteristics of Patient and Caregiver Participants

We collected self-reported demographic characteristics, including age, race, ethnicity, geographic location, health insurance, educational attainment, gender identity, sex assigned at birth, sexual orientation, relationship status, cancer diagnosis, and partner’s gender identity, using a survey administered in REDCap and completed by the participants.

#### 2.3.3 Quantitative Analysis of PROMIS Measures and Demographic Characteristics

Demographic data were tabulated overall and across groups. PROMIS measure responses were converted to t-scores consistent with the PROMIS scoring manual. These are based off a population mean of 50 with a standard deviation of 10, where a higher t-score represents a higher presence of the measure of interest. Average t-scores were compared between patients and caregivers within dyads, and between SGM and H/C patients across dyads, using a linear mixed effects model that accounted for the repeated measurements made within participants, and within dyads, using random intercepts. We assessed adequacy of the model by performing an analysis of the residuals to ensure that they conformed to required assumptions. Statistical significance was declared for two-sided p-values less than 0.05.

#### 2.3.4 Qualitative Data Collection and Analyses of Patient and Caregiver Interviews

We conducted qualitative semi-structured interviews to elicit information regarding the intersection of sexual orientation, gender identity, and cancer care. To respect the differences inherent in the four participating groups (i.e., SGM and H/C patients and their two groups of caregivers), we developed four semi-structured interview guides: an SGM patient guide, an SGM caregiver guide, an H/C patient guide and an H/C caregiver guide. The first two guides had three distinct categories of questions: (1) life as a member of the SGM community; (2) experience of a cancer diagnosis/treatment; and, (3) support systems/coping mechanisms. The second two guides had the same questions as the SGM guides, but did not include questions about SGM-specific experiences. We pilot tested the interview guides with advisors from the SGM community. We selected advisors who were cancer patients and informal cancer caregivers. They also held positions as leaders of SGM organizations, healthcare providers, and SGM community advocates. We revised the interview guides according to feedback received.

Participants selected their preferred interview modality, videoconference, *via* telephone, or an in-person interview using COVID precautions. Interviews lasted 1-2 hours, were audio-recorded and transcribed for analysis. Based on initial hand coding of three de-identified, semi-structured interviews, members of the team developed a codebook and three primary coders (EB, SAJ, and SR) undertook question-level dual coding, thematic analysis using the *dedoose* research platform. The larger team met for iterative analysis, comparing and contrasting codes, grouping similar content or meaning into broader themes, describing linkages, at individual levels, dyadic levels, and cross-dyadic (SGM and H/C) levels. Recurring themes were highlighted, and presented in the following “Results” section. Qualitative findings were compared to quantitative findings to triangulate dominant qualitative themes with key domains identified in the quantitative survey. Patient and caregiver recommendations to improve care were mapped using an ecological model to organize next steps for research and intervention development.

## 3 Results

### 3.1 Characteristics of Patient and Caregiver Participants

In total, 34 individuals participated in this study (n=19 SGM, n=15 H/C), see **Table 2**. The average age of participants was 68 (SD=13). The majority was white and non-Hispanic (94%), and lived in an urban area (91%). Only 1 patient reported being uninsured. Most completed graduate or professional school (73%), and described their gender identity as woman (65%). Twenty-nine percent of participants reported their sexual orientation as lesbian, 12% identified as gay, and 47% as heterosexual. The majority of participants indicated their relationship status as married (71%). Half of the patients had a diagnosis of breast cancer, followed by colorectal (13%), lung (9%), ovarian (9%), and pancreatic (9%).

**Table 2:** Patient and caregiver demographics.

	Dyad		Role		Total Sample
	*SGM*	*H/C*	*Caregivers*	*Patients*	
N	19	15	16	18	34
Age [Mean (SD)]	66.8 (10.2)	68.8 (15.8)	71.5 (12.2)	64.4 (12.8)	67.7 (12.8)
Racial Identity [N (%)] *					
American Indian or Alaska Native	0 (0)	1 (6.7)	1 (6.3)	0 (0)	1 (3.0)
Hispanic or Latino	1 (5.6)	0 (0)	1 (6.3)	0 (0)	1 (3.0)
White	17 (94.4)	14 (93.3)	14 (87.5)	17 (100)	31 (93.9)
Are you of Hispanic, Latino or Spanish Origin? [N (%)] **					
No, not of Hispanic, Latino or Spanish origin	15 (88.2)	15 (100)	14 (93.3)	16 (94.1)	30 (93.8)
Yes, Mexican, Mexican-American, Chicano	1 (5.9)	0 (0)	1 (6.7)	0 (0)	1 (3.13)
Yes, another Hispanic, Latino, or Spanish origin	1 (5.9)	0 (0)	0 (0)	1 (5.9)	1 (3.13)
Do you consider where you live either an urban or rural area? [N (%)]: Urban	16 (84.2)	15 (100)	14 (87.5)	17 (94.4)	31 (91.2)
What type of insurance did you have when your first started cancer treatment? [N (%)] ***					
Private/Commercial	7 (43.8)	6 (40)	5 (38.5)	8 (44.4)	13 (41.9)
Medicare	5 (31.3)	5 (33.3)	4 (30.8)	6 (33.3)	10 (32.3)
More than 1 type of insurance	3 (18.8)	4 (26.7)	3 (23.1)	4 (22.2)	7 (22.6)
Uninsured	1 (6.3)	0 (0)	1 (7.7)	0 (0)	1 (3.2)
What is the highest level of schooling you have completed? [N (%)] *					
Some college or vocational school	0 (0)	3 (20)	2 (12.5)	1 (5.9)	3 (9.1)
Completed a 4 year College degree	3 (16.7)	3 (20)	5 (31.3)	1 (5.9)	6 (18.2)
Graduate or Professional School	15 (83.3)	9 (60)	9 (56.3)	15 (88.2)	24 (72.7)
I describe my gender identity as: [N (%)]					
Woman	13 (68.4)	9 (60)	8 (50)	14 (77.8)	22 (64.7)
Man	3 (15.8)	6 (40)	6 (37.5)	3 (16.7)	9 (26.5)
Transgender Man	1 (5.3)	0 (0)	1 (6.3)	0 (0)	1 (2.9)
Genderqueer	1 (5.3)	0 (0)	1 (6.3)	0 (0)	1 (2.9)
Other	1 (5.3)	0 (0)	0 (0)	1 (5.6)	1 (2.9)
What sex marker is on your original birth certificate? [N (%)]: Female	16 (84.2)	9 (60)	10 (62.5)	15 (83.3)	25 (73.5)
I describe my sexual orientation as: [N (%)]					
Lesbian	10 (52.6)	0 (0)	5 (31.3)	5 (27.8)	10 (29.4)
Gay	4 (21.1)	0 (0)	1 (6.3)	3 (16.7)	4 (11.8)
Queer	2 (10.5)	0 (0)	1 (6.3)	1 (5.6)	2 (5.9)
Bisexual	1 (5.3)	0 (0)	0 (0)	1 (5.6)	1 (2.9)
Heterosexual	2 (10.5)	14 (93.3)	8 (50)	8 (44.4)	16 (47.1)
Other	0 (0)	1 (6.7)	1 (6.3)	0 (0)	1 (2.9)
What is your current relationship status? [N (%)]					
Single	5 (26.3)	0 (0)	1 (6.3)	4 (22.2)	5 (14.7)
Married	11 (57.9)	13 (86.7)	12 (75)	12 (66.7)	24 (70.6)
In a domestic partnership	3 (15.8)	0 (0)	2 (12.5)	1 (5.6)	3 (8.8)
Widowed	0 (0)	1 (6.7)	1 (6.3)	0 (0)	1 (2.9)
Divorced	0 (0)	1 (6.7)	0 (0)	1 (5.6)	1 (2.9)
What was your primary or original cancer or tumor-type diagnosis? [N (%)] **					
Breast	7 (41.2)	9 (60)	7 (50)	9 (50)	16 (50)
Colorectal	2 (11.8)	2 (13.3)	2 (14.3)	2 (11.1)	4 (12.5)
Lung	1 (5.9)	2 (13.3)	1 (6.3)	2 (11.1)	3 (9.4)
Non-Hodgkin Lymphoma	2 (11.8)	0 (0)	1 (7.1)	1 (5.6)	2 (6.3)
Pancreatic	1 (5.9)	2 (13.3)	1 (7.1)	2 (11.1)	3 (9.4)
Ovarian	3 (17.7)	0 (0)	2 (14.3)	1 (5.6)	3 (9.4)
Other	1 (5.9)	0 (0)	0 (0)	1 (5.6)	1 (3.3)
Partner's Gender Identity [N (%)] ***					
Woman	10 (62.5)	6 (40)	10 (62.5)	6 (40)	16 (51.6)
Man	3 (18.8)	7 (46.7)	4 (25)	6 (40)	10 (32.3)
Transgender Man	1 (6.3)	0 (0)	0 (0)	1 (6.7)	1 (3.2)
Genderqueer	1 (6.3)	0 (0)	1 (6.3)	0 (0)	1 (3.2)
Other	1 (6.3)	0 (0)	0 (0)	1 (6.7)	1 (3.2)
I prefer not to answer	0 (0)	2 (13.3)	1 (6.3)	1 (6.7)	2 (6.5)

1: Two sets of summaries are presented, one within the patient/caregiver dyads by SGM vs. H/C, and one within the SGM vs. H/C groupings by patient/caregiver status.

2: * 1 missing value; ** 2 missing values; *** 3 missing values.

### 3.2 Patient and Caregiver Quality of Life

We summarized the scores from these instruments with means, standard deviations, within each of the four groups, and estimated differences of interest, with their corresponding standard errors and p-values (see **Table 2**). Two of the caregivers did not complete the QoL questionnaire.

**Figure 1:**
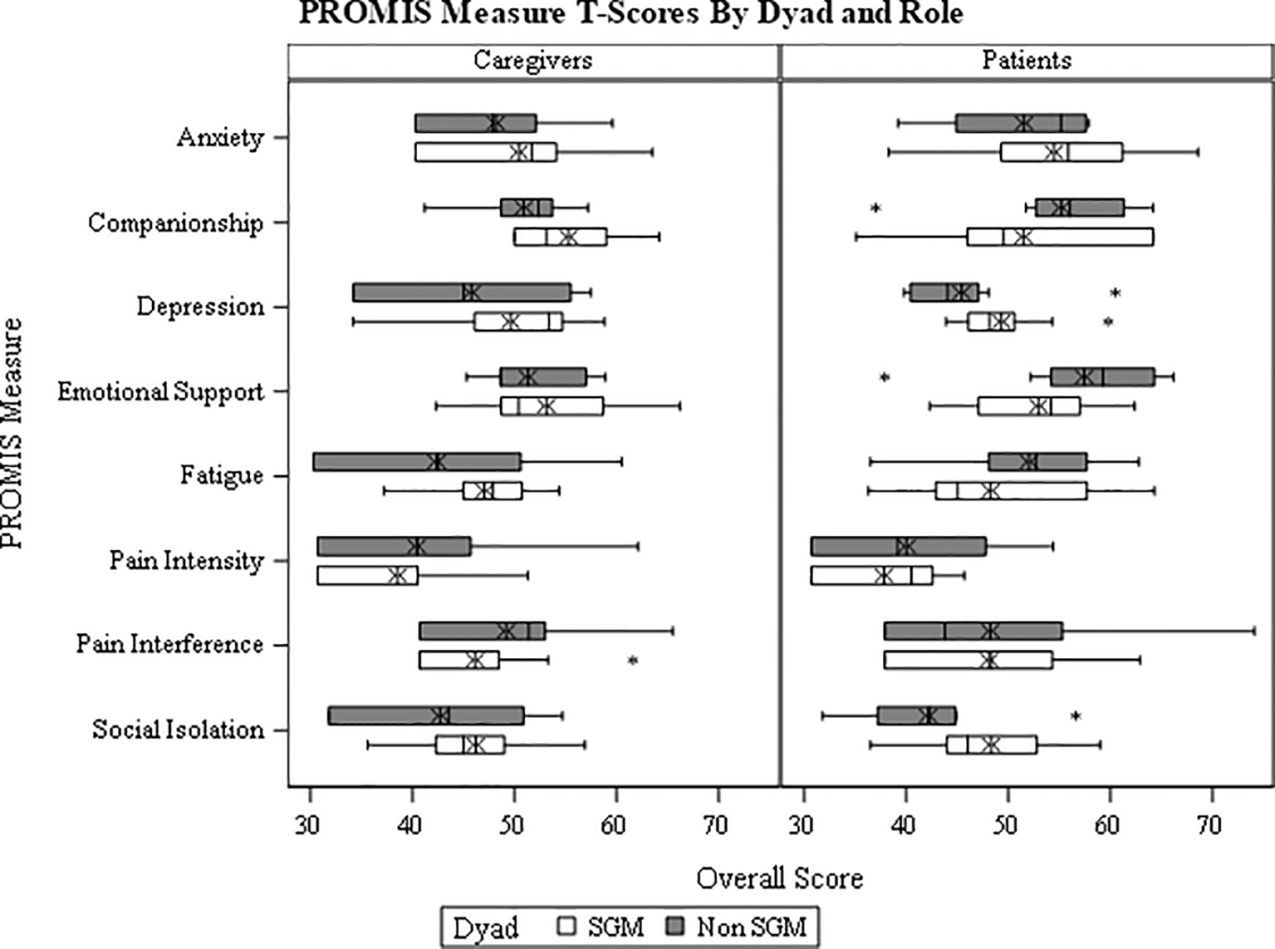
PROMIS Validated Measure Self-Reported Quality of Life for SGM Patients and Caregivers Compared to Heterosexual, Cisgender Patients and Caregivers.

We made between-group comparisons of interest while accounting for the paired nature of dyads, and for the multiple PROMIS scores obtained from each participant, using a linear mixed effects model. Analyses of the residuals indicated this choice was appropriate. Results of the linear mixed effects models suggest that patients and caregivers had significantly different profiles of PROMIS QoL responses (p=0.038), and that the differences in these overall profiles did not reach statistical significance between SGM and H/C patients (p=0.334). See **Figure 1**. In spite of a significant difference in the aggregate QoL profile between patients and caregivers, no per-measure differences were identified as being statistically significant (see **Table 3**; all p>0.05). Estimates of between-group differences that are of potential interest for future study include: patients tended to report more fatigue and anxiety than non-patients did [model-based difference (standard error [SE]) = 4.84 (2.89) and 3.65 (2.70), respectively]; SGM participants tended to report higher depression and social isolation than H/C counterparts [model-based difference (SE) = 3.75 (2.49) and 4.88 (2.76), respectively].

**Table 3:** Summary of PROMIS instrument scores.

	Patients		Caregivers	
	SGM	Non SGM	SGM	Non SGM
Fatigue	48.3 (9.5)	52.0 (8.2)	47.1 (6.0)	42.4 (11.5)
Pain Interference	48.1 (8.5)	48.3 (13.1)	46.1 (7.5)	49.2 (9.2)
Pain Intensity	37.8 (6.4)	40.1 (9.3)	38.5 (8.5)	40.4 (11.5)
Anxiety	54.5 (8.8)	51.6 (7.5)	50.4 (8.6)	48.2 (6.7)
Depression	49.3 (4.8)	45.4 (6.8)	49.6 (8.5)	45.8 (9.3)
Emotional Suppot	53.0 (6.9)	57.5 (9.3)	53.1 (7.5)	51.3 (5.0)
Social Isolation	48.3 (7.3)	42.1 (7.4)	46.2 (6.8)	42.7 (9.5)
Companionship	51.5 (11.0)	55.2 (8.6)	55.3 (6.0)	50.9 (5.5)

### 3.3 Qualitative Findings From Semi-Structured Patient and Caregiver Interviews

Qualitative interviews comparing the perspectives and experiences of SGM and H/C dyads highlight differing experiences of cancer care, structures of social support and coping, and allow for analysis of care delivery in order to characterize gaps in SGM cancer care. Qualitative interview questions specific to SGM experiences of Sexual Orientation and Gender Identity (SOGI), and SOGI related stigma and discrimination, provide relevant context to the lived realities and cancer care of SGM patients and caregivers in the study. The following representative quotes are presented in the following sections: 1) SGM patient and caregiver experiences stemming from sexual orientation and gender identity; 2) SGM and H/C patient similarities and differences in experiences of cancer care; 3) SGM and H/C caregiver similarities and differences in experiences of providing support for cancer patients; and 4) Patient and caregiver suggestions to improve cancer care. Subthemes in each section highlight recurrent and significant topics identified through iterative analysis. An outline of sections, subthemes, and queried and/or contrasted participants, is presented in **Table 4**. Quotes are edited to remove verbal pauses and repetition to increase reader accessibility. Quotes are coded by interviewee role and SOGI (i.e. PT = Patient, CG = Caregiver, SGM = Sexual and Gender Minority, H/C = Heterosexual, Cisgender).

**Table 4:** Summary of thematic qualitative findings.

Themes	
3.3.1 SGM Patient and Caregiver Experiences	
Subthemes	SGM patients and caregivers experienced anti-SGM stigma and discrimination leading to “minority stress”
	All were concerned about potential stigma and discrimination in healthcare settings
	Previous stigmatizing experiences contributed to medical mistrust in cancer care
	SGM caregivers articulated feelings of stress more acutely then did their H/C counterparts
	Single SGM patients experienced loneliness, isolation, and lacked community support
	SGM patients and caregivers are resilient and use coping strategies during cancer treatment and care
3.3.2 The Need to Improve SGM Cancer Care	
Subthemes	Oncology staff and providers lack SGM cultural competence training and SGM medical knowledge
	Oncology teams are inconsistent in the inclusion of SGM caregivers in patient decision-making
3.3.3 Heterosexual, Cisgender Patient Experiences	
Subthemes	Comfort at the cancer center
	Ability to be critical of cancer care
	Differing patterns of support
	Distinctions in the articulation of the cancer experience
3.3.4 Overlapping Themes in SGM and H/C Patient and Caregiver Interviews	
Subthemes	All patients appreciate oncologists, nurses and cancer care navigators
	All patients rely heavily on caregivers
	There is insufficient support for caregivers regardless of SOGI
3.3.5 Patient and Caregiver Suggestions to Improve SGM Cancer Care	
Subthemes	Ask patients and caregivers about SOGI
	Train staff and providers in cultural humility and communicative competency
	Gain knowledge of SGM sexual health relevant to cancer treatment
	Identify and/or offer tailored support services for SGM cancer patients
3.3.6 Patient and Caregiver Suggestions to Enhance Support for All Caregivers	

#### 3.3.1 SGM Cancer Patient and Caregiver Experiences Relating to Their Sexual Orientation and Gender Identity

##### 3.3.1.1 SGM Patients and Caregivers Experienced Anti-SGM Stigma and Discrimination Within Their Lived Experiences

SGM patients and caregivers in this study, all of whom have lived through periods of intense social change, described both challenges and opportunities relating to their SOGI and membership in the SGM community. All articulated examples of anti-SGM stigma and discrimination within their lived experiences. The majority described periods when their sexual and gender identities were not accepted in the dominant society. More than half had moments of fear and insecurity stemming from their marginalized minority status. A caregiver explained:

*“…the biggest challenge is never feeling like I was quite accepted or loved enough in my family … so that would be my biggest existential crisis, feeling like there’s something wrong with me.”* - SGM CG-7

A bi-sexual patient told us, *“Attractions to women weren’t options when I was younger. I hadn’t had really good role models. It was scary to me.”* - SGM PT-7.

Hiding sexual orientation due to employment restrictions magnified these fears. A lesbian caregiver told us: *“I’m 80 years old, so I go way back. Being gay in the 60s and 70s was really scary, especially in the military. If you even had a friend who was gay, you could be discharged. It was very difficult to hide that. Every day, you’re living a lie. Every day, you live in fear.”* - SGM CG-3

A lesbian caregiver explained that fear and stress, “alters our behavior sometimes, it drives decisions.” – SGM CG-5 One lesbian patient told us, “*I’ve always looked over my shoulder. I’ve always monitored what I say and how I behave. I don’t walk around with a sign. When I’m with someone, I don’t even know that we’d hold hands. I’m always careful about my safety. I’ve never felt totally safe. That’s just the way it is*.” - SGM PT-2

Patients and caregivers reported how such experiences continued in their current lives. Several mentioned verbal assaults, two losing jobs due to their SOGI status, and one couple felt discriminated against when purchasing a home. Two patients lost custody of children. Gender nonconforming participants felt “*policed*” when using public restrooms.

*“Anytime I was in a public space, I felt like there was somebody there who thought it was their job to not let me use the bathroom…’You can’t go in that bathroom. That’s the wrong bathroom.’…It was stuff like that. I still sometimes find myself holding it when I could just go.”* - SGM CG-1

*“I have certainly experienced situations where I felt questioned, “What are you?” When I travel, a lot of places, I’ll get sir’d. I’m actually comfortable with that. What I think is uncomfortable is when people get it, like, “Actually, you’re not a guy. I’ve never felt threatened, but I’m someone who definitely gets looks going into the women’s bathroom.”* - SGM PT-10

All SGM patients and caregivers discussed their individual lives in relation to broader social and structural situations. Many lost friends during the AIDS Epidemic. Others experienced fear of police, hostile politicians, and anti-SGM policies. Others fought against marriage exclusions. All who were born female discussed the role of sexism in their lives. Participants acknowledged that these broader issues compounded SGM specific stressors. One patient explained how she internalized homophobia and stigma:

*“…the part that’s toxic is you always have to wonder … Just the fact that you even have to think about it is kind of where the toxicity comes from. It’s almost an internal problem because you have no way of actually knowing, unless somebody comes up and calls you a dyke to your face and punches you. People are smarter than that, usually. It’s really insidious—it’s just a factor. It’s an added stress factor in all of your interactions.”* - SGM PT-5

##### 3.3.1.2 SGM Patients and Caregivers Experience Stigma and Discrimination in Healthcare Settings

SGM patients and caregivers encounter stigma and discrimination in healthcare settings and medical institutions. A lesbian patient shared how she and her wife experienced medical discrimination when having their son:

*“When we had (son’s name), …the attorney general rule said that if you’re married you can fill out the birth certificate, and the other same-sex parent can go on the certificate. We filled out all that information … When we actually got his birth certificate, it was completely blank. The nurse had just not put in (partner’s) information.”* – SGM PT-5

One lesbian patient described her strategy for coping with healthcare discrimination, saying, *“I’ve tried to navigate my way through discrimination by leveraging my white, straight-passing privilege. It’s a little bit harder without the hair … At the hospital, there’s this kind of implied “We’re supportive.” I think they’d like to think they’re more supportive than what they are. I think they put on a good veneer. They do things that are surface-level supportive, but it doesn’t feel as heart-connected.”* - SGM PT-7

The patient’s wife and caregiver, picked up on the same sentiments, telling us of an experience where she sensed that her relationship was unacceptable to a hospital administrator:

*“[wife’s name] and I were sitting on a bench at the hospital. I was kind of leaning in toward her, and the [administrator’s title] went by, and I could feel her discomfort. I think she tried hard not to feel— ‘Ugh.’ Maybe that could be anyone … It could be any affection. I don’t know. [But] I suspected that she didn’t feel comfortable because we were two women, and it was near a public area.”* - SGM CG-7

##### 3.3.1.3 SGM Patients and Caregivers Found Resilience and Belonging in the SGM Community

Even with such challenges, the majority of those in the study found belonging and resilience from being part of the SGM community. Many described members of the broader SGM community as “*family*.” One women said, “*In the lesbian community, I have a sense of belong and affirmation*.” - SGM CG-9 Another told us, ““*Community is a really big source of, if not empowerment, then inspiration. To see people who are struggling or who are dealing with or have dealt with intractable issues or traumatic issues. It is amazing … here in [city name], community is really strong*.” - SGM PT-10 For others, empowerment came from community activism. One lesbian patient explained:

*“I worked, for 11 years, in the HIV and AIDS community as a therapist at an AIDS agency. That was wonderful because, at the beginning of it, AIDS, two-thirds of the agency were folks who were gay, lesbians. It was very empowering. Then, being in a relationship with the love of my life for 12 years, that was empowering.”* - SGM PT-4

A gay male patient said:

*“I’ve been out now for, gosh, how many years is it? I’ve also worked in the gay community, so I’ve been aligned with the community for a long time and been an activist. After all of these years, it’s kind of in my gay DNA. I feel pretty empowered. In an interesting kind of way, discrimination is a reaffirmation of a very important part of my identity.”* - SGM PT-6

Although such experiences occurred outside of cancer care, they informed the lives, behaviors and coping strategies of SGM cancer patients and caregivers.

#### 3.3.2 SGM and H/C Patient Similarities and Differences in Experiences of Cancer Care

##### 3.3.2.1 SGM and H/C Patients Appreciated the Cancer Center and Oncology Care Teams, Although SGM Patients Emphasized the Need to Develop Trust With Their Physicians More so Than Did Their H/C Counterparts

Although both SGM and H/C patients appreciated the care given at the cancer center, their experiences differed. H/C patients overall described situations where they felt instantly welcomed. One patient told us, *“I can’t say enough good things about UNM Cancer Center. We were given all the time; we were given wonderful explanations. They have always been welcoming and supportive and interested in more than just my cancer. I think that’s really important.” -* H/C PT-1

Two others mentioned patient navigators. One patient described, *“What I found extremely wonderful at the cancer center was the navigator. She would just show up. There she was this little ray of sunshine. I would be there for a test, and she’d give me a hug and a couple words….that was special.” H/C PT-4 A second claimed, “I felt welcomed. [name] was my navigator. He met me at the door, and I had talked to him on the phone. He was great! He took us all around, showed us things, and then he took us up to my appointment.”* – H/C PT-6

SGM patients did not describe such feelings of instantaneous welcome, instead, focusing on staff and provider efficiency and communication that led to feelings of trust. As one genderqueer patient said, “*I was super impressed. I felt like they were efficient, that they knew what they were doing. The expertise was really high. I have just a high bar for what I think is good practice.”* – SGM PT-10

One lesbian patient described the moment when she began to trust her oncologist:

*“I felt a lot of hope in meeting with [Dr.’s name] … because that’s when the switch flipped … she said one thing about having an 85% response rate to treatment. That just totally shifted my paradigm in the moment. Then she said, ‘I still don’t have enough information to give you any idea about what this looks like for you.’ I feel like she is a straight shooter … all of those things make me feel positive.”* - SGM PT-7

Another explained that she appreciated her oncologist’s responsiveness to her questions, even though had to bring up up issues relating to sex and sexuality that she felt were important to her care:

He was very focused, very responsive to my questions. I talked to *him about sexuality, sex, and the sexual experiences and how the anti-estrogen pill was affecting me … Another time, we talked about lubrication. Of all the questions that they asked you, none of them were about your sexual life and your sexual functioning. He’s on some committee, and he actually told me he brought up, to the committee, that that’s not asked. Now they’ve been negotiating how the doctor should address that issue. I was really excited that I had some influence and that he is committed to it and is working on it. He said most patients don’t bring it up, but it clearly would affect their lives.* – SGM PT-4

H/C patients likewise called attention to oncology team communications. One women told us, *“I met with [Dr.’s name] and just really, really liked her so much and liked her approach and her bedside manner. I felt like she was a good fit.” - H/C PT-2 Another patient said, “My doctor is fantastic! She takes the time. She is busy. I know that. But if I have questions, if something’s off, out of the ordinary, or whatever, she always takes the time and answers it. That means a lot. It’s important that they’re listening.”* - H/C PT-8

##### 3.3.2.2 SGM Patient Critiques About Cancer Care Delivery Resulted From a Lack of Culturally Appropriate Service Delivery, Whereas H/C Patients Took Issue With Gaps in Patient Provider Communication in Response to Care Needs

Although both SGM and H/C patients offered critiques of various components of cancer, lingering concerns they chose to emphasize differed. On the one hand, SGM patient concerns were rooted in discomfort caused by a lack of SGM cultural competence and heterosexist medical assumptions. A lesbian patient described an uncomfortable situation with the breast reconstruction team:

*“I was weirded out by my interactions with [Dr.’s name] and how he interacted with [wife’s name] as well. In the very first meeting, they have me stand in front of a green screen … a resident … or junior attending … took pictures of my breasts with his iPhone, which I think is probably not standard protocol. It just felt very slimy, the whole thing. They were talking about what my boobs would look like after the fact. I just remember being very uncomfortable; the whole thing just made me uncomfortable … it felt very much like an old boys club.”* - SGM PT-5

A genderqueer patient expressed discomfort with the assumptions made about breast reconstruction:

*There were assumptions that were made about how important the body part of breasts were to me. It just felt like every person who inhabits a female body is really going to care about breast conservation. And I really didn’t. I probably would have really appreciated being asked, “How do you feel about that part of your body? What’s your relationship with your breasts?” I think it could be any part of the body that had cancer, but for people who are in a female body, the breasts are one of the most charged body parts that there are.* - SGM PT-10

H/C patients, on the other hand, took issue with gaps in patient provider communication and response to care needs. One women described how she was informed of her cancer:

*“…the worst of all the experiences, one morning, we’re sitting here at breakfast, the phone rings. 7:00 am. A voice I can hardly hear or understand because there is a terrible connection says, ‘This is [Dr.’s name] and you have breast cancer.’ I was furious! Why do you call somebody up and do that?”* - H/C PT-1

Another patient suggested:

*Of all my experiences with UNM Cancer Center, there is only thing I would call negative, or not up to the standards of everything else I’ve come to expect. I’ve talked to two people on that side of the office, but in both cases, they were very nice. They helped them. They gave me information, but I asked for more information and never got it. I called about the status of things and never got a call back. That’s the way it goes sometimes.* - H/C PT-7

One woman told of a crisis resulting from medication:

“I got my infusion and then I was trying to take the pills and I *was having a really hard time and feeling very nauseous … I tried to get a hold of an oncologist after hours and I got a nurse who said there was no oncologist on call. That was absolutely horrible … The nurse couldn’t even pronounce the drug that I was on, and then couldn’t connect me to an oncologist … she was out of state!”* - H/C PT-5

##### 3.3.2.3 SGM Patients Felt Were Often Uncomfortable About Their Loved Ones’ Inclusion by Providers, a Sentiment Not Shared by H/C Patients

SGM and H/C patients also experienced staff and provider inclusion of their caregivers and families in very different ways. All H/C patients felt that their husbands, wives, and children were included in care. Patients told us,

*“My husband is such a rock. He came with me to every single appointment up until radiation because he couldn’t come into the radiation room. At that point, I had already had six months of chemo, a lumpectomy, a mastectomy. My sister came to one, and my best friend came in from Houston to hold my hand for my first and last chemo. I have an incredible network of support.”* - H/C PT-2

*“My husband was with me throughout. And our son came with me because my husband is not medically savvy. Even though I am, I am the patient, and it still was an emotionally difficult time for me. So, to be objective and to really hear everything that was happening, our son is very capable and took a lot of notes and was certainly a support for us to get through this all. We believe life hits you, lots of things, but it’s a journey we’re in together. Cancer is a family diagnosis I feel strongly about that, and that they needed and had a right to know what was happening with us.”* - H/C PT-4

One SGM patient, whose partner is a transman, offered a similar sentiment:

*“…it’s hard to retain the information we get in any kind of doctor’s appointment, and he was there with me for that. I knew I would not be able to remember everything, which is what actually did happen. He is the holder. However much he remembers today, he is the holder of a lot of the information … It makes me feel sad for people who don’t have partners or close relationships … I’m blessed.”* - SGM PT-9

Other SGM patients in the study had differing feelings regarding caregiver inclusion in their care. One couple chose to hide their partnership. They told us, “*They think [Wife’s name] and I are sisters, and lots of times we just let them think that … we just let them believe whatever they want to believe, or we just say we’re friends kind of thing. Some of the times we just smile and nod.”* - SGM PT-3

Another woman spoke of the discomfort she and her wife experienced with the care team:

*“They knew that she was my person. They just didn’t know what to call her. [That] one thing was always a little awkward. Is she your wife? Is she your partner? Is she your spouse? There were a number of times where it was just fumbling for the right verbiage. I think the easy way to do that is to just ask at the beginning, like, “How should I refer to you?”* - SGM PT-5

Another couple explained how they discussed their relationship with the doctor prior to care to ensure that she would be able to work with them:

We asked [Dr.’s name]. ‘Are you OK working with a lesbian couple?’ Because neither of us really trusted that she, or any medical professional is, because people have all kinds of stuff. There is a lot of religiosity even among doctors and healthcare *professionals, where they have biases; those biases come out. We just wanted to hear explicitly that she was—then she went into this whole thing of how she—in her undergraduate degree, she did this research on HIV. It was kind of like she was telling us that she was really queer-friendly, or at least kind of LGBT-friendly. We wouldn’t have had to do that if we were a straight couple -.* – SGM PT-10

##### 3.3.2.4 SGM and H/C Patients Had Differing Patterns of Social Support

Similarly, SGM and H/C patients indicated differing patterns of social support. H/C patients were likely to rely on family. Patients told us:

My family is just 100% with me. My wife our two kids. We *never had anything even remotely like this affect our family. This is really something where my family just came together. In that first meeting and for several meetings, all four of us were there. Everybody was involved and jumped right on and did everything they could and still are.* - H/C PT-7

*I was very blessed. I have a wonderful home to be in and enough money to make myself comfortable and I had somebody to care for me … I really didn’t feel that I could have gotten through the chemotherapy without help. But my husband, and my son were very helpful, and neighbors and friends brought groceries and helped us stay in our home which was very important.* - H/C PT-4

SGM patients were much less likely to rely on family. Partnered SGM patients relied heavily on their spouses and partners. Others found support through friends, work colleagues and neighbors. One woman explained, *“We just decided, ‘We’re going to enlist our village.’… my kids’ schools was supportive. We had friends that would bring us food. My parents were disasters. It wasn’t surprising. It’s hard when your parents aren’t interested in your treatment at all.”* - SGM PT-5

Three of the four single SGM patients experienced loneliness, isolation and lacked community support. One man told us:

*“The biggest challenge for me has been socially, now that I’m older and single. Most other gay folks that are my age are in relationships. Especially as an older gay man who doesn’t fit into the young and beautiful kind of images that are so often desired, there’s a certain amount of loneliness … There’s an odd thing that has happened in terms of my own sense of myself and my identity. I think of myself as a cancer patient. In a similar way as being gay, [cancer] informs my life and decisions … it exacerbates my feelings of loneliness.”* – SGM PT-6

An SGM woman expressed similar feelings:

I had one friend that moved to Maui. She was the one person I *could go out and have a beer with. Or we’d go and eat together, take walks, whatever. I miss that. Everybody else is in couples … I did see two women [names friends – in a partnership]. I saw them last week, and it was wonderful. I didn’t want to go home. I stayed there so long that I got caught when they shut down the freeway. It took me like two hours or two and a half hours to get home. But I wouldn’t have traded it because I got to see these two people.* - SGM PT-2

##### 3.3.2.5 Cancer Center Support Services, While Underutilized by Both SGM and H/C Patients, Were Effective When Engaged

The majority of SGM and H/C patients did not take advantage of cancer support services such as counseling, support groups, financial assistance or nutritional counseling. Reasons for the lack of service use varied. Reasons for underutilization of support groups for SGM patients centered around “not wanting to identify with the disease” -SGM-PT-9, not wanting the group to “bring me down” -SGM PT-3, and a lack of SGM specific groups. One patient described her attempt to go to a non-SGM specific cancer support group in the community:

*“There’s a group … for women earlier than 40, that are diagnosed with breast cancer. I went to that once or twice. I think that that was the only time that I ever felt out of place because I was gay. They were talking about their husbands. I don’t know. It felt very young, straight, not my people.”* – SGM PT-5

H/C patients generally suggested that they received support from family and friends, and did not need support groups. One patient explained:

*“I know there are people we can talk to, counselors and stuff. I haven’t really used them, because I do have a lot of support from my family. My parents have been very good. I talk to them a lot. My husband has been amazing, and my sisters. My mother came out for ten days and then my sisters came out.”* – H/C PT-5

Two patients did access support services and found them to be effective. A single lesbian woman who was having financial difficulties stemming from cancer care described:

*“I was struggling with fatigue and some anxiety about the future. Every treatment, the co-pay was $576, every three weeks. I thought, since I was still working, there wouldn’t be any help, but a friend of mine, another therapist, kept nagging me, ‘Call the oncology social worker.’ I finally called her and asked for help, and lo and behold, there’s a Patient Advocate Foundation, and they are paying my co-pays for the chemo. I felt like I won the lottery. That was so wonderful.”* – SGM PT-4

An H/C patient had a positive experience with nutritional *counseling as well.*

*“I had a phone conversation with the nutritionist, and that was very helpful. I wanted to know is there something I should be doing during chemotherapy? During chemo radiation? To prepare for surgery? She was talking about foods that can be good for maintaining weight. She also told me during chemo radiation I might have to eat more because it’s a healing process, too. One of the first nurses that I spoke to, said that I shouldn’t eat fresh, uncooked vegetables and fruit during chemotherapy, because of the issues with immunocompromised people. The nutritionist said, ‘Actually, this neutropenic diet is kind of old school. We now think that it’s important to eat fresh fruits and vegetables.’ She explained what to eat and how to clean it, so that was helpful.”* – SGM PT-5

##### 3.3.2.6 SGM and H/C Patients Used a Number of Coping Strategies During Cancer Treatment and Care

Patients in the study utilized a variety of coping mechanisms to combat the stresses of cancer. The coping mechanisms appeared to have little to do with SOGI and more to do with individual patient preferences, ability and life circumstances. Many suggested that maintaining a positive attitude was key. One SGM patient said, “*My psychology is pretty chill. I’ve had a longtime Buddhist practice. I’m sort of someone who doesn’t get thrown off. Part of it is practice.” –* SGM PT-10 An H/C patient likewise said that she tends “to have a positive outlook on the world.” – H/C PT-1 Another patient told us: *Cancer is a moment, a terrifying moment, but it’s trying to keep it in perspective, taking some control over what you have control of, being hopeful, and perhaps living more in the moment. –* H/C PT-4

Still one H/C patient felt the need to “switch hats” and “take care of herself,” a difficult challenge for women and mothers who are “caregivers by nature.” -H/C PT-2. While an SGM patient needed to get tough. She said, *I’m not all that positive about the world as it is, but I took things on as a challenge and as a, “We’ve got to get through this.” My nephew sent me a card, “Cancer is tough, but you are tougher.* – SGM PT-3 Another SGM patient refused to identify with the disease:

*“The thing I remember clearly was [turning to partner] and I said, “No fucking way. I am not identifying with this disease.” I’ve been around a long time. I know people over-identify with illness and I’m just not taking that route. I am going to take the route of, I have this disease, I’m going to get treatment, and that’s it! I think that actually helped me.”* – SGM PT-9

Some patients coped through humor. One woman recounted a moment with her sister:

*“I just felt really, really scared that I was going to die soon. After I had the hysterectomy. I was crying and I said, ‘I really always thought I was going to live to be old,’ and [my sister] looked at me and she started laughing and she said, ‘You are old.’ (Laughter.) ‘Okay.’ And we were able to laugh in the middle of all of this.”* – SGM PT-12

One woman watched movies with her husband, saying, “My *husband and I watched a lot of funny movies: oldies, Johnny Carson Show. I still laugh over I Love Lucy. Television was great. That kept me—and we could do that together.”* – H/C PT-4

Some relied on alternative therapies to support their cancer care journeys including acupuncture, massage, energy work, and herbal remedies. One patient told us, “*I love acupuncture. It works for me. Energy work, all of it works for me.”* SGM PT-2 A few relied on physical activity and exercise. One patient admitted, however, that she had “less ideal coping mechanisms”, telling us: *“I’ve probably been eating more than I should. I’ve probably been drinking more than I should.” -* SGM PT-4

#### 3.3.3 Caregiver Similarities and Differences in Experiences of Providing Support for Cancer Patients

##### 3.3.3.1 SGM Caregivers Did Not Always Feel Comfortably Acknowledged by Oncology Staff and Providers, an Experience Not Shared by H/C Caregivers

All H/C caregivers, like the patients discussed above, described positive feelings about the cancer center and acknowledgement of their position as caregiver in the lives of the patient they supported. One caregiver told us:

“*The first visit, it was all four of us, our two children and me. We all went. That was such a surprisingly pleasant experience … they had a cellist playing in the lobby. I thought, ‘Wow, this is really something.’ The whole family, the support. I’ve always felt that from UNM. It was very helpful and positive. We were all part of the initial treatment plan, when [Dr.’s name] was telling us what was going to happen next and how things were going to go. From the minute, you walk in … whoever you encounter is very nice and pleasant.”* – H/C CG-7

Yet, SGM caregivers had very different experiences. Oncologists and oncology teams’ deficits in knowledge were apparent in their inconsistent inclusion of SGM caregivers in patient meetings and patient decision-making processes. A caregiver told us:

*“I didn’t feel seen. I kept trying to connect with (name of doctor) in a way that would validate me. I said, ‘I work in a hospital; I know the system. I lost my sister to cancer and I was her caregiver.’ But I never got recognized as somebody….it wasn’t worth fighting to try to impress my point. I just held onto my observation. I walked out of there feeling like I did everything but stand on my head to get acknowledged. It made me so angry.”* – SGM CG-7

Another caregiver recounted a “strange” interaction with her partner’s doctor during the discussion about breast reconstruction where she felt the physician may have been *responding to H/C contexts.*

*“She was asking [partner’s name] about if she would want her breasts reduced, something about breast size or breast reconstruction. I just remember her looking at me and she was like [adamant voice], ‘This is her decision to make.’ I was like, ‘Uh, duh, of course,’ and then I was like, ‘What the hell? Are you used to men saying shit? That’s not me!”* SGM CG-P-2

One caregiver reflected on the lack of engagement she felt from staff at the cancer center:

*“I don’t remember anyone asking me how I was at the cancer center or offering any support … Actually, I remember saying that if there had been a group, I would have gone. I was partly just kind of curious because I was like, “What do I not know? I’m just showing up as best I can.” But if it was a group it would have to be overtly queer-friendly. I don’t think I would have wanted to go and have to gender switch. I wouldn’t want to have to be on guard at all.”*—SGM CG-2

##### 3.3.3.2 SGM Caregivers Articulated Acute Feelings of Stress More Frequently Than Did Their H/C Counterparts

SGM caregivers were more apt to indicate that caregiving was stressful than were their H/C counterparts. One SGM caregiver recounted:

*“I have medical PTSD. Everybody doesn’t like hospitals, and I have a special pathological relationship to it and feeling to it. That was just really hard. I wanted so badly to be a good partner, and be there and be reliable and helpful. Every time we would go into these settings, it would just send me into orbit. Every single day we went to the cancer center for those appointments it was just like, ‘Ugh,” just ringing my bell all the time. Surgery … she had a lumpectomy and I was like, ‘Oh, God.’”* – SGM CG-9

Another SGM caregiver related:

*“It was all-consuming. I pretty much ignored work and let some of my peer managers help me out and filled in for me when they needed to. I wasn’t there a lot; I took as much leave as I possibly could … [it was] a huge emotional toll, but it was definitely grief and it was not something I had ever experienced before in any way like that. It’s certainly the absolute hardest time in my life, no question about it.”* - SGM CG-5

Still another told us, “It took over my entire life … Every minute I thought about it. ‘How could I do this better? How could I talk her into eating? How could I get the compression hose on easier? How long was this going to…?” I was getting up at night sometimes and going in to make sure that she was alive.” - SGM CG-3

H/C caregivers, the majority of whom were male spouses, described their caretaking duties differently, as more of an expansion of roles and responsibilities. One caregiver said, “*It didn’t drive me into deep depression or anything. It didn’t change anything; it just was different and there was different activities, and different focus on things*.” -H/C CG-1 Another caregiver explained:

*“I try to think positive. It was really tough at first. We live in a two-story house, and when we came home [wife’s name] barely made it upstairs to our bedroom. Early on when she needed a lot of attention—I ran myself ragged running up and down. I lost a lot of weight, which was good. That became my day-to-day life. Now, I also never gave up golfing.”* - H/C CG-6

The one H/C caregiver who described extreme stress due to caregiving duties was the mother of the patient. She told us, “It’s hard to keep her comfortable because after chemo her head is very warm … we put an icepack on … she’s also had a problem with mouth sores. That’s the hardest thing. I had no idea what to do for that. Physically, sometimes it’s very difficult.” – H/C CG-2

#### 3.3.4 Patient and Caregiver Suggestions to Improve Cancer Care

At the end of the interviews, all patients and caregivers were offered the opportunity to provide suggestions to improve cancer care. SGM patients and caregivers, although satisfied with their oncology care once trust had been established and treatment was underway, offered the following suggestions.

##### 3.3.4.1 Ask About Patients About SOGI

Patients desired staff and providers to directly address SOGI. A lesbian patient said, *“It’s been challenging to come out to certain people—doctors.” – SGM PT-4. A gay patient added, “It’s such a big and integral part of my life, and that it’s never come up.”* – SGM PT-6

##### 3.3.4.2 Train Oncology Staff and Clinicians in SGM Cultural and Communicative Competency

Staff and physicians often seemed challenged with how to interact with SGM patients and their caregivers. Patients suggested that “cultural competence training” saying, “*We need training for the doctors and front end people. Not all of them. We have run into some remarkable clinic people, doctors and nurses. It’s just difficult. The best thing you can do is bring the best of yourself to it. Some of us have to work on that.*” SGM CG-8. Another caregiver suggested that it should not be a single training, saying, “*To make sure it’s inviting for people who are queer, transgender, gay and lesbian, it has to be real. I know businesses have tried to go through a training, and then put up a sticker, but that has to be lived. It’s not just about training.*” – SGM CG-10

##### 3.3.4.3 Gain Knowledge About SGM Sexual Health Relevant to Cancer Treatment

Lack of discussion about SOGI prevented candid patient and provider conversations about the effects of cancer care. One lesbian patient found a therapist who was a breast cancer survivor to see for her “lack of sex drive” following cancer *treatment. – SGM PT-9. Another lesbian patient suggested:*

*“I definitely would encourage the oncology team to be more informed. Be open about sexual orientation and sex life, because that’s an important part of our health—physical and emotional. My doctor went online to check things out. He should already know or have recommendations, like a CBD oil or a CBD lubricant (to counteract “aging” effects of taking an anti-estrogen pill).”* – SGM PT-4

##### 3.3.4.4 Identify and/or Offer Tailored Support Services for SGM Cancer Patients

Few local support services exist to assist SGM patients and caregivers. One lesbian patient asked for, “a support group that was actually lesbian, and meets in person.” -SGM-PT-4

A caregiver who had worked with those diagnosed with HIV and AIDS, suggested that cancer care might use mentorship model to enhance care for SGM patients and caregivers. He explained:

*“They paired up people who are going in and being told, ‘Your test came out positive’ with another person who had been through it and was already alive ten years later. …the mentor had already been through this experience, had the knowledge about logistically what was going to happen, but also held the emotional knowledge, ‘I’ve been where you are. I know how fearful this is. You’re going to make it through.’”* – SGM CG-9

An SGM patient mentioned that [name of another cancer center where she received a second opinion] offered a similar program.

*“Basically, you fill out a form where you check off some characteristics, like what kind of cancer you had, how old you are, and I think sexual orientation is on there. But if not, you could add it. They just put you in the ranks until somebody has a similar diagnosis and offer you up as a community partner.”* - SGM PT-5

##### 3.3.4.5 SGM and H/C Patients and Caregivers Interviewed Offered Suggestions to Assist Informal Cancer Caregivers

Patients and caregivers had myriad suggestions on how the Cancer Center could better include them in their loved one’s cancer journey. One SGM caregiver provided an idea for people at the onset of care who may be identifying their primary caregiver(s):

*“I would hope that you [could] come up with a variety of profiles of what caregiving might look like. I think sharing those stories with people broadens their perception of what caregiving can be about … [This could be helpful for] people that need care so that they can pick appropriate caregivers and they can begin to identify what are the range of their needs, and who could provide that? Those discussions are really important at the beginning of diagnosis.”*- SGM CG-6

Another caregiver expressed a desire for organized classes on a variety of topics including cooking for cancer patients and addressing side effects their loved one might experience:

*“If they would establish some kind of formal training that would tell us what’s going to happen when we get home and how to deal with that without just getting into it and trying to find your way through it. If they had something like that, I guess it would be like a caregiver support group … Maybe even cooking classes … I don’t know. Anything that we could get involved in.”*- H/C CG 6

Interviews with all patients and caregivers, both SGM and H/C, documented needs for enhanced caregiver supports. Caregivers wanted more explicit information about their loved ones’ treatment side effects and tools that they may use to mitigate side effects. Caregivers wished to receive a “roadmap” to help them navigate each step of their loved one’s cancer journey.

## 4 Discussion

This multi-methods work presents SGM patient and caregiver perspectives of cancer care, contrasting those to the experiences of H/C patient and caregivers. We documented and compared these experiences to identify gaps or misalignments in cancer care delivery that contributed to disparate experiences and outcomes for SGM patients and their caregivers. We intend to use these findings to develop interventions that will improve SGM cancer care.

Quantitative findings using PROMIS validated measures call attention to the complexity of stress and distress in the lives of SGM cancer patients and caregivers. Although this is a pilot study, with a small population, and our findings are not generalizable to the entire SGM population, they do show that SGM patients and caregivers in our study have higher perceived levels of depression, anxiety, and social isolation when compared to H/C patients and caregivers. These findings, although not statistically significant, contradict the Hutchcraft, et al. study which used PROMIS measures finding higher relative odds of psychological distress among bisexual cancer survivors, but not among lesbian cancer survivors (50). While few studies have used PROMIS measures to assess SGM QoL to date, studies have assessed psychological health in SGM cancer patients and survivors. A systematic review by Gorden, et al. indicated SGM mental health disparities in male cancer patients, but not in women (51). Studies by Jabson and Bowen (52) determined that SGM women had higher levels of perceived stress, yet studies by Kamen et al. (17) and Boehmer et al. (53, 54) that indicate little to no differences in perceived stress although some differences with regards to anxiety and/or depression between SGM and H/C women cancer survivors.

As in studies by Kamen et al. (55) and Hsieh et al. (56) ours points to the role of role of minority stress as a contributing factor in cancer care for SGM patients and caregivers. Even among a predominantly white, middle class participant population, SGM patients and caregivers recounted numerous experiences of stigma and discrimination at personal, community, and national levels that contributed to minority stress and medical mistrust. Although these experiences predated cancer diagnosis, they contextualized cancer care encounters. Feelings of medical mistrust, minority stress, and distress were heightened for patients and caregivers prior to entering the cancer center and during initial encounters with staff and physicians across various cancer care teams (i.e., oncologists, radiologists, plastic surgeons). Minority stress in SGM patient and caregiver cancer care experiences is evident when comparing accounts of SGM dyads to those of H/C dyads for whom considerations of acceptance and trust are less urgent. As examples, in initial cancer center/cancer team encounters: *“I can’t say enough good things about UNM Cancer Center. We were given all the time … They have always been welcoming and supportive and interested in more than just my cancer;” -*HC-PT-1, contrasted with*, “We asked [Dr.’s name]. ‘Are you OK working with a lesbian couple?’ Because neither of us really trusted that she, or any medical professional is, because people have all kinds of stuff. There is a lot of religiosity even among doctors and healthcare professionals, where they have biases; those biases come out. We just wanted to hear explicitly that she was.” –* SGM PT-10. Also within caregiver feelings of inclusion in cancer care, *“The first visit, it was all four of us, our two children and me … I thought, ‘Wow, this is really something.’ The whole family, the support;” -* H/C CG-7 compared to, *“I didn’t feel seen. I kept trying to connect with (name of doctor) in a way that would validate me … But I never got recognized as somebody….it wasn’t worth fighting to try to impress my point … I walked out of there feeling like I did everything but stand on my head to get acknowledged. It made me so angry.” –* SGM CG-7

Importantly, due to the size and limitations of our participant sample, our pilot had low representation of gender minorities. We can only report, therefore, on the experiences of one genderqueer patient and one transgender caregiver. Even so, both gender-nonconforming participants, identified in the text of the results/findings, clearly indicated moments where minority stress was exacerbated due to prior experiences of mis-gendering, bathroom policing, and in the case of the caregiver prior medical PTSD that made it challenging for him to support his partner during her cancer care.

Our qualitative findings, like previous studies by Kamen et al. (55) and Hsieh et al. (56) indicate that although minority stress is chronic for many SGM individuals, it may not be a consistent barrier to cancer care for all patients. SGM patients and caregivers are incredibly resilient, drawing strength and empowerment from membership in the SGM community, as well as from caregivers, social support networks, and healthy coping strategies. In the majority of cases, SGM patients and caregivers coped with familiar patterns of minority stress, and were able to focus on their cancer care, once connections with staff and providers were secured, and comfortable communication and trust established. Overall, SGM patient and caregiver experiences, even those that had challenging moments, resulted in positive cancer care experiences.

### 4.1 Recommendations to Improve SGM Cancer Care

**Figure 2:**
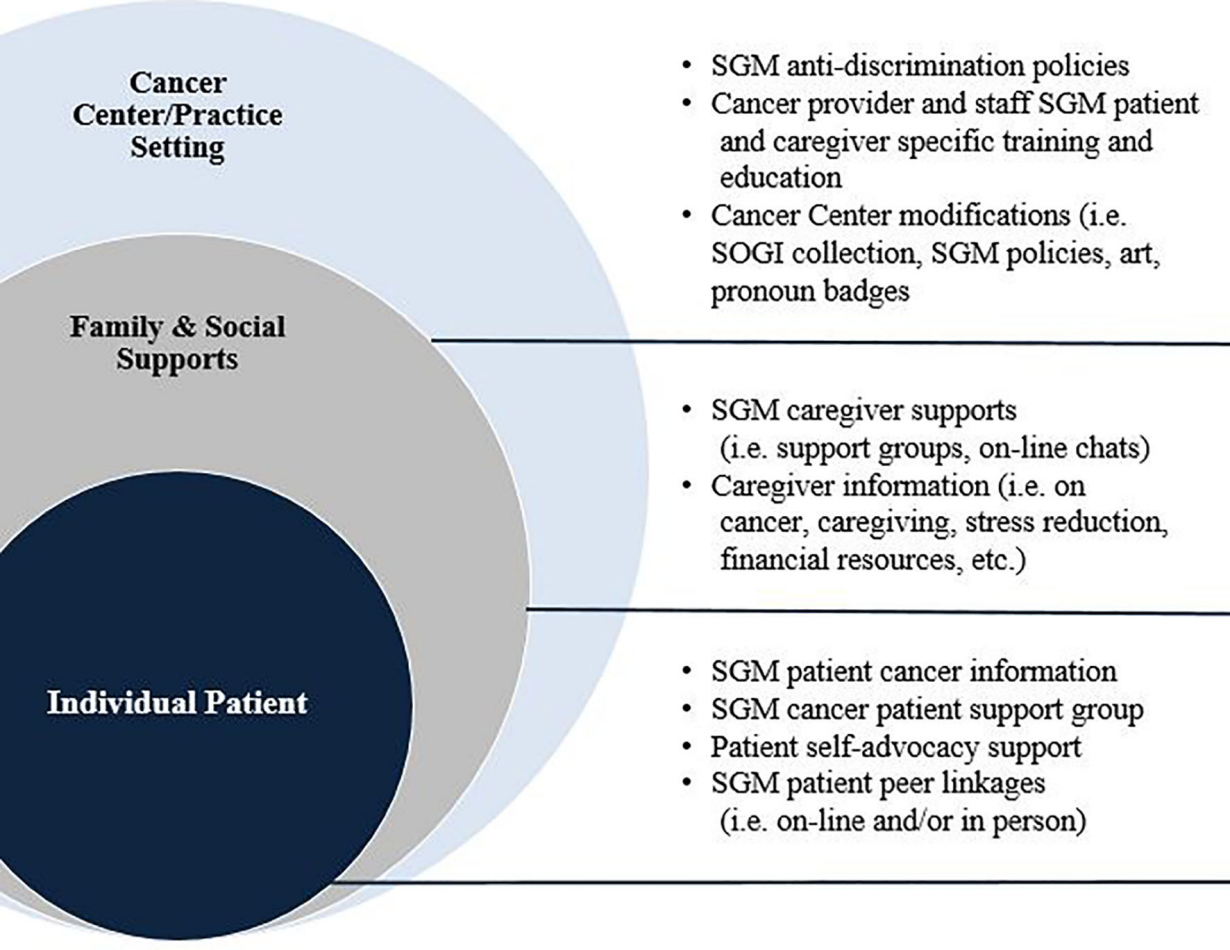
An Ecological Model of SGM Patient and Caregiver Suggestions to Decrease Gaps in Cancer Care.

These pilot findings align with scientific recommendations by Huelsman et al. (57) and Kano et al. (58) underscoring the need to include SGM specific programming and SGM affirmative practices across cancer center levels to enhance care for SGM patients and their informal cancer caregivers. Drawing from patient and caregiver recommendations that demonstrated relational gaps in optimal cancer care for SGM patients and their caregivers, we employed ecological theory, to map suggestions at multiple healthcare levels (**Figure 2**): (a) cancer center/organization, (b) administrator, provider and staff, (c) caregiver and social support, and (d) individual SGM cancer patient (59). At the cancer center/practice setting level, immediate and regular training for staff and clinicians in SGM culturally competent communication and care provision would facilitate quick connection between SGM patients and caregivers and oncology care teams to alleviate medical mistrust and stress/distress stemming from minority stress. Providing visual cues around the cancer center would also facilitate patient and caregiver feelings of acceptance. Provider and staff training would increase SOGI data collection, and decrease heterosexist models of cancer care that fail to account for SGM recognition and care preferences, previous traumas, and alternative family/caregiver structures. At the family and social support level, our study emphasizes the need to develop culturally tailored support for SGM caregivers. Although no two cancer journeys are the same, development of a general packet of information targeted at caregivers could ease stresses associated with caregiving. At the patient level, our study highlights the need to develop and provide SGM tailored supports through groups and/or one-on-one formats, increase patient self-advocacy, and enhance patient information. Care should be taken to support single SGM patients who may be experiencing loneliness and isolation, or for whom support is lacking hindering access to care and positive recovery from cancer. Likewise, all interviewed mentioned the importance of nurse/patient navigation as critical to their overall experience at the cancer center. However, provision of such services did not appear consistent throughout patient care, leading to patient and caregiver frustration. This issue could be addressed by implementing a model where patient/nurse navigation occurs at regular intervals throughout the entire cancer journey.

### 4.2 Study Limitations

We received funding for this study at the onset of the COVID-19 pandemic, a factor that delayed the start of the study due to research closures, and caused a shift in methodology from face-to-face interaction to electronically (Zoom) mediated encounters. Furthermore, changes in cancer care delivery hindered our ability to recruit a diverse (i.e. racial/ethnic, socioeconomic, and rural) participant population by limiting most in-person cancer center visits to patients undergoing active treatment, increasing utilization of telehealth for rural and follow-up patients, and drastically limiting caregiver entrance to the cancer center. Therefore, we relied on self-recruited patients, who came into the cancer center for treatment, and saw our flyers. The majority of our study population was non-Hispanic white, had college degrees, and were not subjected to serious financial hardship as a result of their cancer diagnoses. It is quite possible that given the uncertainties of the pandemic, national attention to police violence against racial/ethnic minorities in the United States (i.e. Black Lives Matter), and contentious political climate, multiply-marginalized patients and caregivers simply did not feel sufficiently safe to participate in research that they may have feared would affect their cancer care. Even so, this pilot revealed relevant information about gaps in care for SGM patients and their caregivers.

## 5 Conclusions

This study demonstrates that prior stigmatizing experiences contribute to minority stress and medical mistrust for SGM cancer patients and their informal caregivers across cancer care encounters. Findings point to specific gaps in SGM cancer patient care, including lack of SOGI discussion, inadequate staff and oncology provider SGM specific knowledge, and insufficient SGM specific patient supports for those who lack social support during cancer care treatment. While we know that consideration of SOGI, SGM recognition, and caregiver preferences are important across all fields of healthcare, these needs are heightened with the stress of cancer diagnosis.

This study also reveals inadequacies in SGM specific support, and overall support services for informal cancer caregivers. Although there were differences between SGM and H/C experiences of cancer treatment, caregivers expressed consensus about the current lack of support and guidance for informal caregivers of cancer patients. Future work should focus on providing caregiver-specific resources in the clinic setting and facilitating support groups for caregivers to network with one another, as well as for tailoring SGM specific caregiver support services.

Overall, this study speaks to the importance of decentering normative assumptions regarding patient and caregiver SOGI, roles and needs, and degrees of social support and isolation, at an individual as well as societal level. Creating safe spaces involves open conversations with patients and caregivers regarding these issues at the outset of treatment and throughout the cancer care experience, along with creating inclusive instruments for assessing physical and mental health, especially in regards to sexual health and quality of life measures. Increasing collection of SOGI data will facilitate provision of care at an individual level and contribute to the development of inclusive initiatives at broader levels. Cancer centers need to do more to acknowledge SGM patient preferences in order to optimize care for underserved SGM cancer patients and their caregivers.

## Data Availability Statement

The original contributions presented in the study are included in the article/supplementary material. Further inquiries can be directed to the corresponding author.

## Ethics Statement

The studies involving human participants were reviewed and approved by University of New Mexico Human Research Protections Office Institutional Review Board (HRRC #20-385). The patients/participants provided their written informed consent to participate in this study.

## Author Contributions

MKa, SJ, SR, DG, EB, and AH reviewed literature on the topic, analyzed qualitative data, and contributed to the writing of this manuscript. MKo and VP provided analysis of quantitative data and writing of the manuscript. TR, ZD, and LM contributed to writing and critical review of this manuscript. All authors contributed to the article and approved the submitted version.

## Funding

This study was funded through an American Cancer Society Institutional Research Grant, ACS-IRG 17-178-22, with additional support provided through the National Cancer Institute funded, University of New Mexico Cancer Center Support Grant, P30CA118100. This research used services provided by the Behavioral Measurement and Population Science Shared Resource and the Biostatistics Shared Resource, facilities supported by the State of New Mexico and a University of New Mexico Cancer Center NCI-funded grant, P30CA118100.

## Conflict of Interest

The authors declare that the research was conducted in the absence of any commercial or financial relationships that could be construed as a potential conflict of interest.

## Publisher’s Note

All claims expressed in this article are solely those of the authors and do not necessarily represent those of their affiliated organizations, or those of the publisher, the editors and the reviewers. Any product that may be evaluated in this article, or claim that may be made by its manufacturer, is not guaranteed or endorsed by the publisher.
